# Are prognostic indices for brain metastases of melanoma still valid in the stereotactic era?

**DOI:** 10.1186/s13014-017-0951-4

**Published:** 2018-01-10

**Authors:** Harun Badakhshi, Fidelis Engeling, Volker Budach, Pirus Ghadjar, Sebastian Zschaeck, David Kaul

**Affiliations:** 1 Department of Clinical Radiation Oncology, Ernst von Bergman Medical Center, Academic Teaching Hospital of Charité - Universitätsmedizin Berlin, 14467 Potsdam, Germany; 2 Department of Radiation Oncology, Charité - Universitätsmedizin Berlin, Campus Virchow-Klinikum, Augustenburger Platz 1, 13353 Berlin, Germany

**Keywords:** Brain metastases, Stereotactic radiation therapy, Survival, Prognostic scores

## Abstract

**Background:**

Malignant melanoma brain metastases (MBM) are the third most common cause for brain metastases (BM). Historically Whole-brain radiotherapy (WBRT) was considered the goldstandard of treatment even though melanoma cells are regarded as very radioresistant. Therapeutic possibilities have fundamentally changed since the availability of stereotactic radiotherapy (SRT), where it is possible to apply high ablative doses in a very precise manner. In this work we analyze prognostic factors of overall survival (OS) after SRT in patients with MBM and evaluate the applicability of popular prognostic indices that mainly stem from the WBRT-era.

**Materials and methods:**

This work is a retrospective analysis of OS of 80 malignant melanoma (MM) patients who received SRT for intracranial melanoma metastases between 2004 and 2014 who had not received prior treatment for MBM in terms of surgery or WBRT. Potential prognostic factors were analyzed using univariable and multivariable analysis. Existing prognostic scores [Graded Prognostic Assessment (GPA), Diagnosis-Specific-GPA (DS-GPA), Golden Grading System (GGS) and RADES] were calculated and tested using log-rank analysis.

**Results:**

Eighty patients, respectively 177 brain metastases, were irradiated. The median survival time from radiation was 7.06 months. Overall, GGS, GPA and DS-GPA were significant predictors of survival. The MM-specific index DS-GPA showed the best *p*-value but did not show adequate division when looking at the two intermediate risk subgroups. RADES did not show any statistically significant prognostic value. In univariable as well as in multivariable analyses a higher Karnofsky-Index, a single BM, and non nodular melanoma (NM) histology were positive predictors of survival.

**Conclusion:**

The existing prognostic scores do not seem to ideally fit for this special group of patients. Our results indicate that the histologic subtype of MM could add to the prognostic value of specialized future indices.

## Background

Brain metastases (BM) are the most common intracranial malignant tumor [1] and 20-40% of cancer patients will develop BM [2]. Characterized by a high probability to metastasize to the brain, malignant melanoma (MM) is the third most common cause for brain metastases. Melanoma brain metastases (MBM) are also the most common cause of death in melanoma patients [3]. The incidence of MM has been increasing by 3–7% per year, which translates into a predicted doubling time of incidence of 10–20 years [4].

In general we have seen an increase in BM in the last decades mainly due to high life-expectancy, better imaging technology and the development of new systemic drugs that in many cases do not adequately penetrate the blood-brain barrier [5].

The median overall survival of a patient with BM is less than 12 months, but OS varies significantly interindividually depending on a plethora of factors like. Karnofsky performance status (KPS), extracranial metastasis (ECM), number of metastases, lesion size and volume, and histologic subtype [5].

Historically the goldstandard of treatment for patients with BM was whole brain radiotherapy (WBRT), a therapy that may improve OS and quality of life (QOL) [6]. However, the potential disadvantages of WBRT include treatment times of several weeks and a neurocognitive decline [7]. Therefore, some authors have tried to implement focal therapies like surgery and stereotactic radiosurgery (SRS) [8]. SRS has been shown to reduce neurocognitive decline while improving QOL without compromising OS [9]. However, it must be mentioned that some authors have shown higher intracranial relapse rates in patients treated with local therapies. Still these higher relapse-rates did not translate into worse OS rates [10]. In routine clinical practice, there is a tendency to use WBRT for patients with higher tumor burden, while focal approaches are offered to patients with higher potential survival rates.

As a consequence, many authors have tried to identify prognostic factors of survival in order to predict the individual patient’s potential survival time. Indices based on such prognostic factors include the Golden grading system (GGS), the graded prognostic index (GPA) the disease-specific graded prognostic index (DS-GPA) as well as the prognostic index published by Rades et al. in 2011 (RADES) [11–14]. While the GPA uses the four factors age, KPS, number of intracranial metastases and ECM, the GGS only uses the three factors age, KPS and presence of extracranial metastases, the MM-specific DS-GPA includes only the two factors KPS and the number of intracranial lesions. The RADES uses age, KPS, ECM and number of BM as well as the interval from primary tumor diagnosis to radiotherapy. These prognostic indices are widely used in clinical practice because most other indices developed to this date use components that are either time-consuming to quantify or subjective (e.g. control of extracranial disease or BM volume) [14].

In this study we evaluated the prognostic value of these four indices in MBM patients who received SRT as primary BM treatment.

## Methods

### Treatment decisions, patient selection and dose regimens

We performed a retrospective analysis of 80 patients (61.3% male, median age: 61 years) with MBMs who had undergone stereotactic radiotherapy as their primary treatment between 2004 and 2014 in our department. Treatment decisions had been based on a vote by an interdisciplinary medical team. Doses applied in multiple fractions were considered fractionated stereotactic radiotherapy (FSRT) and high single doses were considered SRS. Larger tumors in close proximity to critical structures were assigned to FSRT, while smaller tumors distant to critical structures were treated with SRS.

### Stratification and variables

Patients were stratified according to age, gender, KPS, histologic subtype, Stage, number of BM, cumulative planning target volume (PTV), highest and lowest BED_10_, synchronous vs. metachronous diagnosis of BM (>1 month after non-small cell lung cancer (NSCLC) diagnosis was considered metachronous), tumor localization, presence of ECM and interval from BM diagnosis to radiotherapy. The prognostic scores, GPA, DS-GPA, GGS and RADES, were calculated and tested using the log-rank test. Prognostic parameters were identified using uni-and multivariable analyses.

Patients with tumor progress could undergo salvage SRS, WBRT or a resection of the BM. Follow-up examinations, including MRI as well as clinical and neurologic examinations were usually performed at 3 months intervals after radiotherapy or if clinically indicated.

### Technical set-up

Patients were treated using Novalis® (BrainLab®) with beam shaping capability, built-in multi-leaf collimator (MLC) and image guidance. Novalis ExacTrac® image guided frameless system enabled us to image the patient at any couch position using a frameless positioning array. All patients received diagnostic contrast enhanced cranial magnetic resonance imaging (ceMRI) and cranial computed tomography (CT) imaging before CT/MRI-fusion planning was performed. The three-dimensional treatment planning system iplanRT® was used. Gross tumor volume (GTV) was defined as the area of contrast enhancement on T1-weighted MRI images, the PTV included a 2 mm isotropic safety margin. The dose was prescribed to the 80% isodose at the PTV margin.

### Formulas and statistics

The biologically effective dose (BED) was calculated according to the following formula, where n is the number of fractions and d the dose per fraction. Following the Linear quadratic model, a value of 10 was used for the *α*/*β*-ratio.

1 $$ BED= nd\left[1+\frac{d}{\alpha /\beta}\right] $$

OS was calculated starting with the first day of irradiation and estimated using Kaplan-Meier method. Subgroups were compared using the log-rank test for univariable analysis and the Cox proportional hazard model for multivariable analysis. A *p*-value of less than 0.05 was considered statistically significant. A p-value of less than 0.1 was considered a trend and was the criterion for the inclusion in multivariable analysis. All statistical analyses were performed using IBM SPSS Statistics 19 (New York, USA).

## Results

### Patients

Patient characteristics are summarized in detail in Table 1. Eighty patients with 177 brain metastases were irradiated. The majority of the patients were male (61.3%) and the median age was 61 years. Most Patients (86.4%) had a good KPS of 70% or higher. Twenty-five percent of patients were already diagnosed Union internationale contre le cancer (UICC) stage IV at the time of cancer diagnosis and 5% of the patients showed synchronous brain metastases. NM was the most common histologic subtype of melanoma (27.5%).

**Table 1:** Characteristics of the 80 BM patients analyzed

Characteristics	No./median (range)	%
Sex (m/f)	49/31	61,3% / 38,8%
Age (y)	61 (16.2 - 88.7)	
KPS		
100	20	25.0%
90	25	31.3%
80	17	21.3%
70	7	8.8%
60	4	5.0%
50	1	1.3%
n/a	6	7.5%
Histology		
SSM	10	12.5%
NM	22	27.5%
LMM	2	2.5%
ALM	2	2.5%
AMM	3	3.8%
occult	13	16.3%
n/a	28	35.0%
UICC stage at diagnosis		
I-III	60	75.0%
IV	20	25.0%
Synchronous BM	4	5.0%
Number of treated lesions		
1	35	43.8%
2	24	30.0%
> = 3	21	26.3%
Cumulative lesion volume (ccm)	2.47 (0.02-41.68)	
BED10	91.1 (39-91.1)	
Fractionation		
SRS	59	73.8%
FSRT	7	8.8%
Both	14	17.5%
Salvage WBRT	8	10.0%
Salvage SRT	23	28.8%
Salvage Resection	1	1.3%
Targeted therapy^a^		
Vemurafinib	2	2.5%
Ipilimumab	4	5.0%
Dabrafenib	1	1.3%

*KPS* Karnofsky performance status, *SSM* Superficial spreading melanoma, *NM* Nodular melanoma, *LMM* Lentigo maligna melanoma, *ALM* Acral lentiginous melanoma, *AMM* Amelanotic malignant melanoma, *n/a* Not available, *UICC* Union internationale contre le cancer, *BM* Brain metastasis, *BED* Biologically effective dose, *SRS* Stereotactic radiosurgery, *FSRT* Fractionated stereotactic radiotherapy, *WBRT* Whole-brain radiotherapy, *SRT* Stereotactic radiotherapy

^a^Targeted therapy given at any point (before and after RT)

Almost half of the patients (43.8%) were treated for a single brain metastasis. Median cumulative lesion volume was 2.47 ccm (0.02-41.68 ccm). Median BED_10_ was 91.1 Gy (39-91.1 Gy).

### Overall survival

Of patients alive at last follow-up, median follow-up time was 7.06 months;

OS rate was 58.2% after 6 months and 29.5% after 12 months.

Univariable and multivariable analysis of prognostic factors is shown in Table 2. Statistically significant factors of favourable OS in univariable analysis were higher KPS, single BM and non-NM histology.

**Table 2:** Univariable and multivariable analysis of potential preditive factors. Factors with a *p*-value ≤0.1 were included in multivariable analysis

Variable	univariable analysis			multivariable analysis		
	HR	95% CI	*p*	HR	95% CI	*p*
UICC stage at diagnosis (<IV vs. IV)	0.63	0.355 - 1.12	0.11			
ECM	1.59	0.69-3.69	0.28			
Synchronous BM	1.56	0.49-5.03	0.46			
Gender (m vs. f)	1.18	0.73 - 1.91	0.5			
highest BED10 (< vs. > = median)	1.14	0.67-1.92	0.63			
Age (< vs. > = median)	1.05	0.67 - 1.69	0.83			
Cumulative BM volume (< vs. > = median)	2.486	1.5 - 4.13	< 0.001 (*)	1	0.94 - 1.06	0.87
KPS (<90% vs. > = 90%)	2.16	1.3 - 3.59	0.003 (*)	3.99	1.78 - 8.96	0.001 (*)
Single vs. multiple lesions	1.68	1.04 - 2.72	0.034 (*)	3.55	1.71 - 7.35	0.001 (*)
Histology (NM vs. other)	1.9	1.02 - 3.52	0.043 (*)	2.26	1.12 - 4.54	0.022 (*)
Interval PT diagnosis to SRT (< vs. > = median)	0.66	0.41-1.06	0.086 (+)	0.79	0.38 - 1.66	0.54

*UICC* Union internationale contre le cancer, *ECM* Extracranial metastasis, *BM* Brain metastasis, *BED* Biologically effective dose, *KPS* Karnofsky performance status, *NM* Nodular melanoma, *PT* Primary tumor, *SRT* Stereotactic radiotherapy

(*) *p*-value ≤0.05, (+) *p*-value ≤0.1

In multivariable analysis KPS, single BM and non-NM histology were significant prognostic factors.

### Prognostic indices

**Fig. 1:**
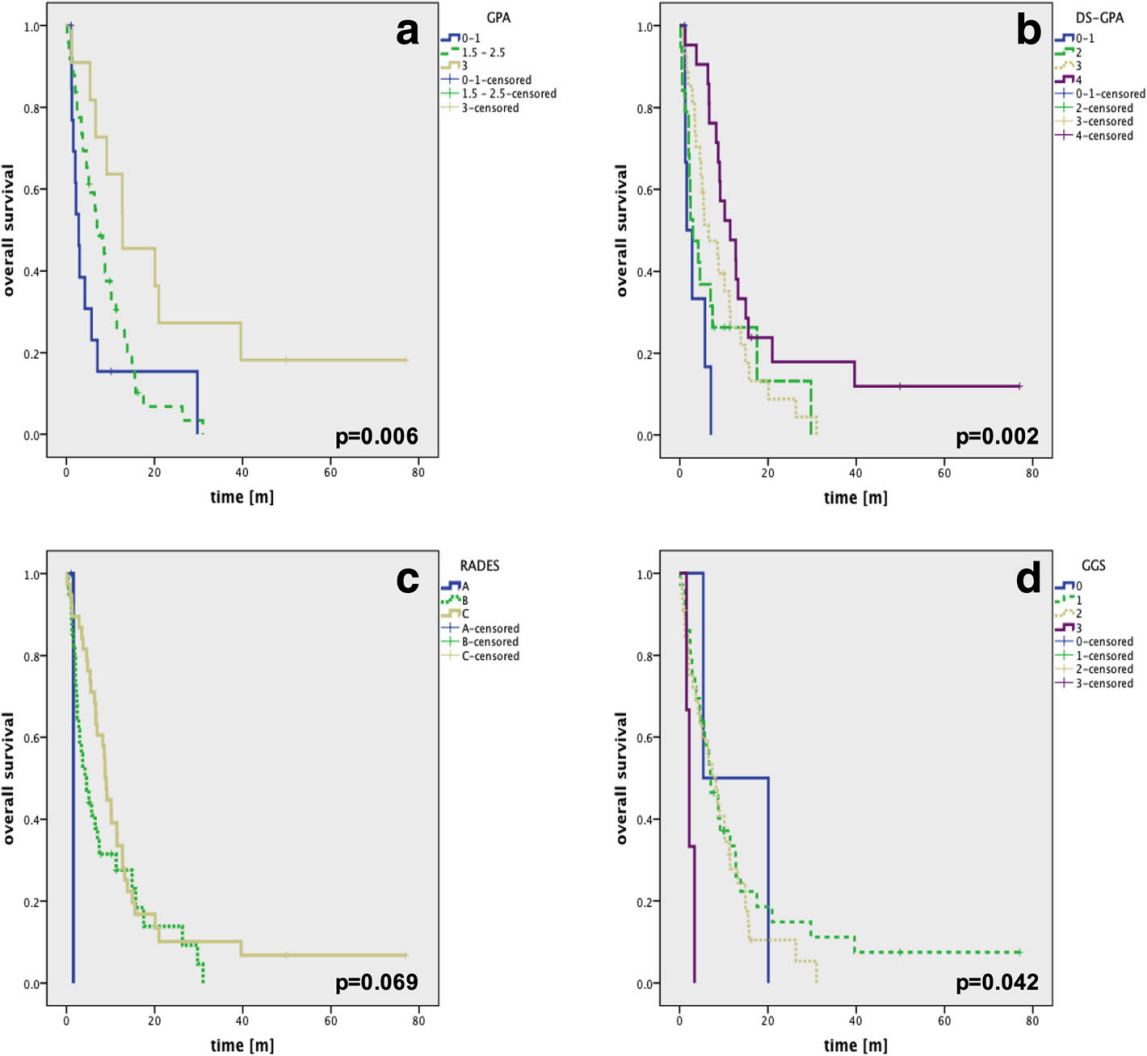
Kaplan-Meier analysis of OS rates. GPA (**a**), DS-GPA (**b**), RADES (**c**) and GGS (**d**). Note that there are only 3 classes in the GPA (**a**) since the cohort did not contain any patients that scored 3.5-4 points on the GPA

An overview of the prognostic value of the four examined indices is given in Fig. 1. In our patient cohort the GPA, DS-GPA and the GGS Index showed significant OS distribution in the Kaplan-Meyer analysis as predictors of survival. The best *p*-value was seen for the DS-GPA. while the RADES gave a poor result. However, none of the four indices gave a full set of non-intersecting survival curves for the respective subgroups (Fig. 1).

## Discussion

The question which BM subpopulation benefits from WBRT or SRS has been a topic of debate in the neurooncologic community for years [15]. A plethora of prognostic indices has been proposed and published. With the help of these indices physicians are now trying to identify subpopulations that might benefit from more focal therapies. In this work we examined four well-known indices in a MM patient population treated with SRT. The indices used here are easy to calculate and do not rely on subjective variables.

All indices examined here (GPA, DS-GPA, GGS, and RADES) use the KPS. A significant predictor in univariable and multivariable analysis of survival in our cohort.

The number of BM was a significant predictor in our univariable and multivariable analysis and is used in the two indices with the best prognostic value (the GPA and the DS-GPA). The GGS does not use this factor but still gives significant prognostic values. The RADES uses the number of BM but fails to give significant prognostic values. Likhacheva et al. [16] were the first to publish a study on prognostic factors in patients with BM who had only received SRS. In their patient cohort the cumulative BM volume was a significant predictor of OS analysis, while the number of BM was not a significant factor. In their publication the authors advise to use BM volume in order to predict OS rather than the number of BM. This is contradictory to our data, where the number of BM was a predictor of OS but BM volume was not.

The factor age was not significant in univariable or multivariable analysis in our cohort. Of the examined indices all but the DS-GPA use the factor age for calculation. Another factor not significant in our cohort was ECM. This factor is used in the GGS, GPA and the RADES index.

The interval from diagnosis to radiotherapy is used in the RADES index. In our analysis we did not identify this factor as significant neither in univariable nor in multivariable analysis.

One factor we did find to be of significant value both in univariable and multivariable analysis was NM histologic subtype, a factor which has not been used before in any of the other examined indices. The primary tumor in NM tumors normally presents with greater thickness than other subtypes of melanoma and therefore metastasizes earlier than other histologic subtypes [17]. However, it should be discussed, that NM histology might actually be a surrogate marker of BRAF status. It is known that BRAF mutations are more common in NM [18] and that BRAF-positive patients survive longer than BRAF-negative patients [19], therefore BRAF status was also included in the new Melanoma-molGPA index published by Sperduto et al. [20].

Looking at the original publications by Sperduto, Rades and Golden, it must be remembered that the patient cohort analyzed here is much more homogeneous in terms of treatment than in the original works. We excluded all patients that had received WBRT or MBM resection as primary BM treatment [11–14].

The fact that the DS-GPA gave the best results can be explained by the fact, that it solely relies on the two most significant predictors of OS in our multivariable analysis: KPS and number of BM. The fact that patients are grouped in four classes in the DS-GPA instead of three classes like the GPA and the RADES makes the DS-GPA even more outstanding.

Kano et al. compared 4 prognostic indices in a cohort of 422 patients who had been treated with Gamma Knife SRS for melanoma BM. The patient cohort included patients who had received WBRT (*n* = 132) or craniotomy (*n* = 65) earlier. The authors compared the recursive partitioning analysis (RPA), the Score Index for Radiosurgery (SIR), the Basic Score for Brain Metastases (BSBM), and the DS-GPA. The authors found the DS-GPA to be the most proved most balanced and prognostic [21].

The limitations of this study should also be mentioned. Firstly, the retrospective nature of the analysis is prone to bias. Secondly, the number of patients may have been too low to find significance of some potential prognostic factors or even of the RADES index itself. Thirdly different regimes of systemic therapy may represent a potential bias.

## Conclusion

Several authors have developed prognostic indices for BM and MBM patients, the ideal index has not been defined yet and further research into alternative approaches is needed. Of the indices examined here, the DS-GPA gave the best results in our analysis in this patient cohort with MBM treated with SRT. Apart from showing the best result in our analysis the DS-GPA is also the easiest index to calculate examined in this work. Our results indicate that the histologic subtype NM might improve the prognostic value of MBM-specific indices.

## Abbreviations

BED: Biologically effective dose
BM: Brain metastases
BSBM: Basic Score for Brain Metastases
CT: Computed tomography
DS-GPA: Diagnosis-Specific-GPA
ECM: Extracranial metastasis
FSRT: Fractionated stereotactic radiotherapy
GGS: Golden Grading System
GPA: Graded Prognostic Assessment
GTV: Gross tumor volume
KPS: Karnofsky performance status
MBM: Malignant melanoma brain metastases
MLC: Multi-leaf collimator
MM: Malignant melanoma
MRI: Magnetic resonance imaging
NM: nodular melanoma
NSCLC: Non-small cell lung cancer
OS: Overall survival
PTV: Planning target volume
QOL: Quality of life
RPA: Recursive partitioning analysis
SIR: Score Index for Radiosurgery
SRS: Stereotactic radiosurgery
SRT: Stereotactic radiotherapy
UICC: Union internationale contre le cancer
WBRT: Whole-brain radiotherapy
